# Low energy KTP laser in oral soft tissues surgery: A 52 patients clinical study

**DOI:** 10.4317/medoral.17428

**Published:** 2011-12-06

**Authors:** Carlo Fornaini, Jean P. Rocca, Elisabetta Merigo, Marco Meleti, Maddalena Manfredi, Samir Nammour, Paolo Vescovi

**Affiliations:** 1 Correspondence: Oral Medicine and Laser-Assisted Surgery Unit – Department of ENT and Cervico-Facial Sciences, University of Parma – Parma (Italy); 2 St. Roch Hospital, Faculty of Dentistry, University of Nice-Sophia Antipolis – Nice (France); 3 Department of Dental Sciences, Faculty of Medicine, University of Liège – Liège (Belgium)

## Abstract

Objectives: Since 1962 laser appliances have been used for soft tissues surgery of oral cavity with significant advantages compared to the traditional instruments: excellent bleeding control, possibility to avoid the use of suture, good patient compliance thanks to a decrease of intra- and post-operative discomfort and biostimulating effect. Unfortunately, the wavelengths so far used have been seen to cause, in association with an excellent ablation capacity, heat damage of the tissues that can decrease healing process and cause a greater discomfort to patients. The aim of this study was to evaluate the laser-assisted KTP laser surgery at low power in terms of characteristics of intervention and patients compliance. Study design: In this study, we describe the application of a new and recently introduced in dentistry wavelength, the KTP laser (532 nm), used with low power (1 Watt - CW), evaluating the time of interventions and, by a Numerical Rating Scale, the intra and postoperative pain. Results: KTP laser used at low power permits to obtain good pain control during operations that were carried out with only a topic anaesthetic (EMLA, Astratech), as shown in VAS tests. Good healing with limited or absent burning areas in treated portion of tissue. Conclusions: These preliminary study allows us to affirm that KTP laser with low parameters permits to perform oral surgery with good pain control and good wound healing. A greater number of clinical cases are however necessary to confirm the result obtained.

**Key words:** Laser, KTP, oral surgery, thermal increase.

## Introduction

Maiman built the first laser equipment in 1960 and after a short period of time, in 1962, this technology was used for the first time in dentistry by Goldman (1). A gradual expansion of laser use as well as the introduction of newer, smaller and cheaper laser wavelengths and devices have been reported (2).

Laser application has been shown to be of particular interest in oral soft tissues surgery thanks to the innumerable advantages that it may offer, compared to conventional instruments. The first wavelengths used in oral surgery was Maiman’s Pulsed Ruby Laser. Argon Laser (488 nm), CO2 (9600 nm), Nd:YAG (1064 nm) and Diode (810 and 980 nm) were subsequently used (3).

Laser equipments may be easily used for surgical treatment of vascular lesions such as haemangioma and lymphangioma, thanks to the possibility to control bleeding and avoid the use of suture. Moreover, patients affected by coagulation alterations as well as hypertension can be treated without specific pharmacological therapy (4).

The effect of biostimulation associated with antibacterial activity improves the wound healing without complications: thus the entire post-operative process may cause little discomfort to the patient (5).

Unfortunately the laser equipments available for dental surgery required high powers to obtain an ablative effect: this in order to achieve a photothermal type of interaction with the target tissue. The side effect is heat damage of the treated tissues, causing an increased discomfort with both intra- and post-operative pain, delay in the healing process associated with oedema of the treated area, limiting the use of this appliance.

To avoid these complications new technological devices have been introduced, such as short shots interspersed by rest periods to allow the tissue “thermal relaxation” (pulsed, superpulsed, chopped modes). In addition it has been reported that these new lasers may determine a decrease in sensitivity perception, less thermal tissue damage, even from the histological point of view (6).

In this study we describe the use of a particular wavelength that, at the moment, is not very widespread in dentistry: KTP laser, is a solid active medium laser emitting in the visible portion of the spectrum (its beam is an intense green light) produced by a special procedure.

Firstly a Diode laser (810 nm) “pumps” energy to stimulate a crystal of Nd:YAG (1064 nm).

Subsequently a crystal of Potassium-Titanil-Phosphate (KTiOPo4) situated between the active medium and the semi reflective mirror in the Fabry-Perot chamber doubles the vibration frequency of the photons and so halving their wavelength and thus emitting the ray of 532 nm (7).

KTP laser has been introduced in medical field thanks to its great affinity for haemoglobin and oxihaemoglobin, becoming very effective in vascular tissues (8). Moreover, in contrast to Nd:YAG laser, in the red oral tissues it is absorbed at superficial tissue level avoiding deep tissue penetration, determining a very safe laser (9).

Even if the cost of this equipment is certainly greater than diode technology, the optic fiber delivery system, that is characterized by a small size, makes it an instrument of great interest.

One of the first uses of KTP in medical field was in Neurosurgery operations (10). At the moment it is widely used in the vaporization of prostatic tumours (11-13) and in otolaryngological surgery of the larynx and epiglottis therapy of papillomatosis. tonsillectomies, naso-farinx tumours and ear-nose small bones operations (14-18).

It has recently introduced to the large intestine surgery (19).

However KTP major use remains vascular surgery and treatment of superficial vascular surgery of the skin, thanks to its affinity for haemoglobin (20,21).

In dentistry it was firstlyl used, with great success, in teeth bleaching, in association with a red hydrogen peroxide gel, complementary colour of the green of this laser ray (22).

Several studies have subsequently reported the efficaciousness in the decontamination of the root channels and periodontal pockets (23,24).

The purpose of our study is to propose the use of this wavelength in the surgery of oral soft tissues using low powers (1 Watt).

Using these parameters, it has been possible to observe that the instrument gives an excellent ablative effect avoiding the use of local anaesthetic injection.

The most interesting caracteristic, however, remains the significant healing capacity of the tissues due to the lack of heat damaging processes (25).

Photochemical effects, responsible for biostimulation and inhibition of pain stimuli, appear at low Power Densities.

Higher values may cause photothermal effects which are present in all the therapies carried out with surgical lasers, causing vaporization, carbonization, burning, coagulation and hyperthermia in the tissues.

The use of this particular wavelength with these low parameters even if in the range of values linked to photothermal effects, is closer to the values defined for the photochemical reactions, responsible of biomodulation.

## Material and Methods

Fifty-two patients (27 males and 25 females; mean age 23 years ± 16) affected by different benign oral pathologies of soft tissues were surgically treated by KTP laser LASEMAR 500 (EUFOTON, Trieste, Italia) using a power of 1 Watt in continuous wave (CW) with a 300 µm optical fibre on contact mode.

**Figure 1:**
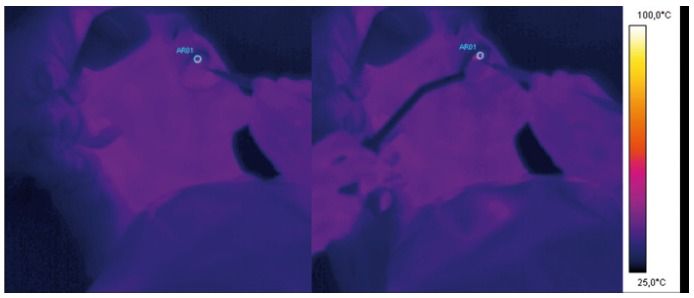
Thermal camera images of an intervention on a retained canine tooth, before (left) and during (right) the surgery.

Different types of surgical interventions have been made ( Table 1) under monitoring with a Thermal Came-ra device (Thermovi-sion – Flyr – Sweden) in order to control the thermal elevations during surgical procedures (Fig. 1).

All the patients underwent a postoperative discomfort evaluation with NRS (Numerical Rating Scale) method reporting the pain they felt during intervention using a number between 1 and 10. The patients were also instructed to complete the same NRS evaluation 3 days after surgery to describe the pain perception during the postoperative period until the 3rd day ( Table 2). 

Each patient was controlled after a week, two weeks and a month. Patients were advised, during healing process, to avoid any acid, salty and/or spicy food, to refrain from brushing the treated area and to use a mouthwash with chlorexidine. An histological specimen was send to a pathologist for all the excised oral lesions in order to evaluate the microscopical characteristics specimens.

Results are expressed as mean and standard deviation.

**Table 1:**
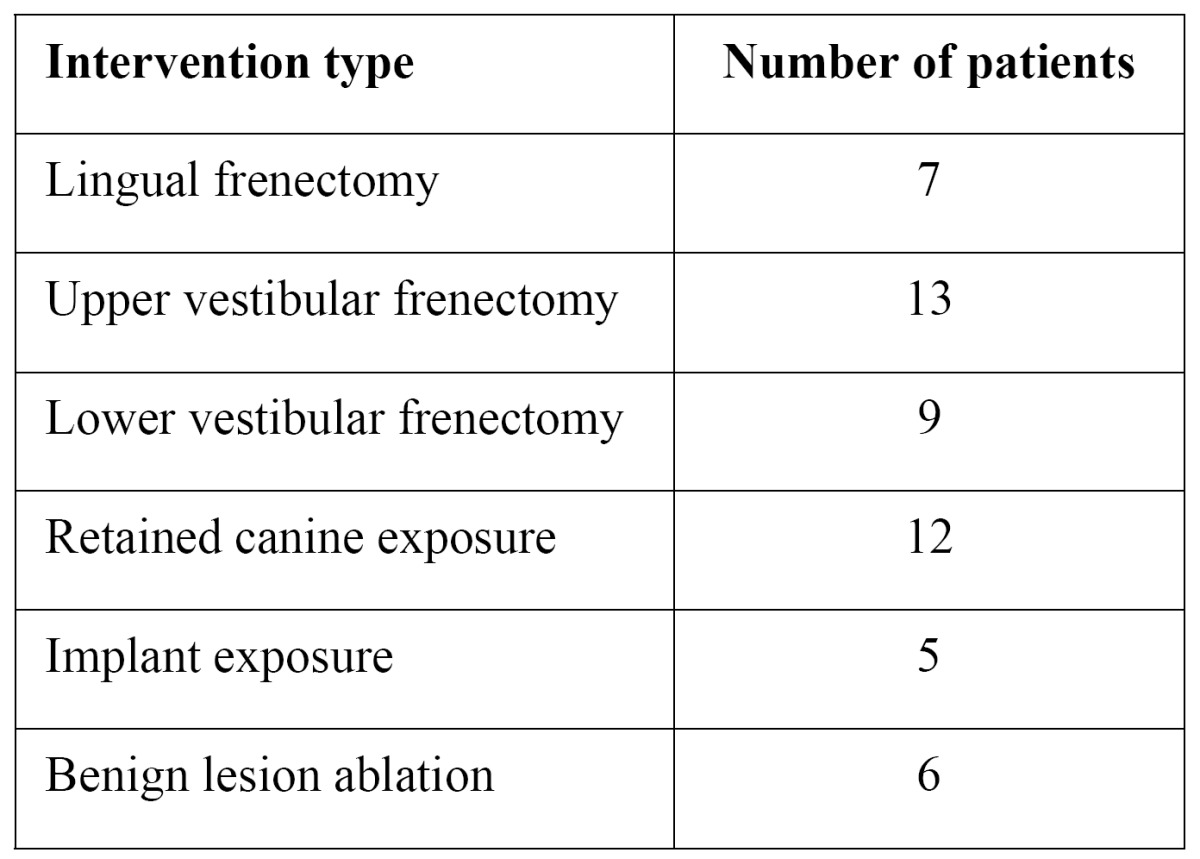
Details about the type of intervention performed.

## Results and Clinical Cases

Thermal elevation recorded with thermal camera device was very limited: mean temperature before treatment was 34,6°C±0,5 and after treatment it was 40,4°C±5,7 with an increase of 5.8°C.

During operation, in which only a topic anaesthetic agent (EMLA, Stretch) was used, patients experienced only a minimum of discomfort and, in any case there was always an absence of pain.

Healing process resulted in all 52 cases by second intention, in that given the haemostasis achieved through the use of the laser suturing was not considered necessary.

Thanks to the possibility to avoid the anaesthetic injection an excellent patients co-operation and compliance were recorded especially in paediatric age or in very anxious adult subjects.

The mean value of surgical NRS was 1.11 and in all the thirty-nine cases it did not exceed the value of 3. Mean value of postoperative NRS was 0.69 with a range between 0 and 3. ( Table 2)

In the post-operative stage eventhough anti-inflammatory drugs were not prescribed and antibiotics were not used, no problem was reported: no oedemas, no infections and no tissue inflammations.

Control examination performed one week after the surgical operation showed a good healing process that was almost concluded after two weeks time and after a month healing appeared completed with the result of a “restitutio ad integrum” situation.

The histopathological evaluation, performed on all the oral benign lesions removed, reported slighty epithelial changes at both nuclear and cytoplasmatic level but did not find at stromal level loss of attachment or collagen denaturation. Vascular changes were observed in presence of blood vessels with signs of coagulation. 

 -Clinical case

**Figure 2:**
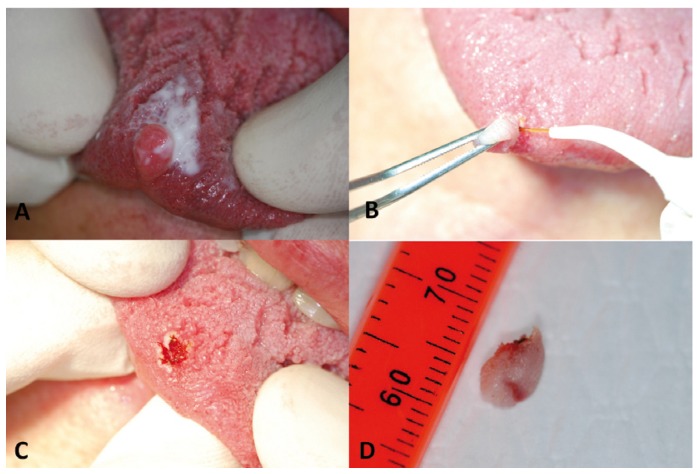
Papilloma of the tip of the tongue: application of topic anaesthetics (Emla, Astra Zeneca) in the interested area for ten minutes (A), surgical laser-assisted removal of the lesion (B), postoperative view of the surgical site (C),macroscopical view of the removed lesion (D).

MR, Female, 60 years old affected by papilloma on the tip of the tongue. After the application of a topic anaesthetics (EMLA, AstraZeneca) in the interested area for ten minutes (Fig. 2), the lesion was removed in 143 sec (Fig. 2) by KTP laser (λ=532 nm), with the following parameters:

Power: 1 W-CW, fiber diameter: 300 µm, contact mode, PD:1244 W/cm2.

**Figure 3:**
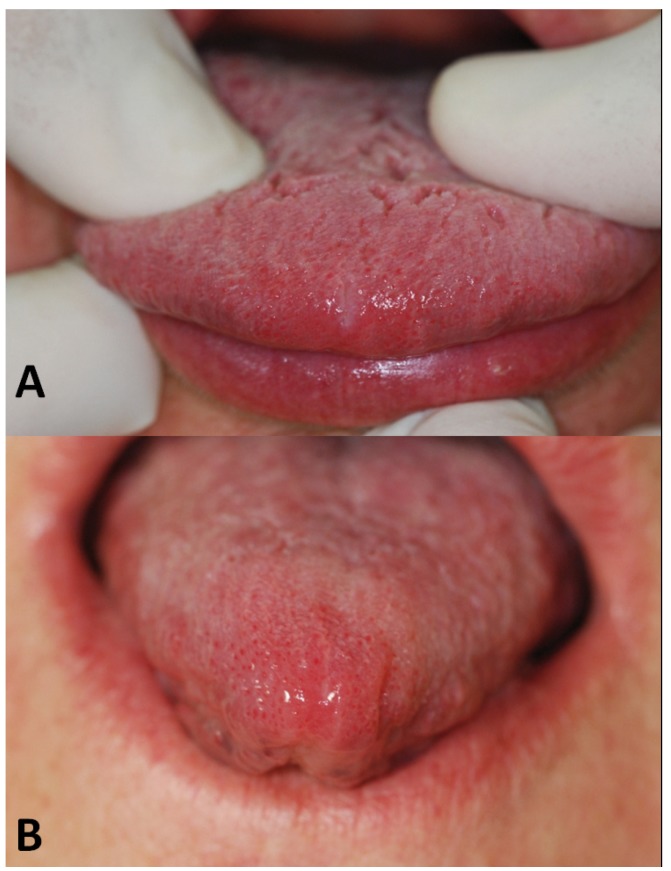
Healing 1 week after surgery (A) and complete healing 2 weeks after surgery (B).

A week after examination showed a good healing pro-cess (formation of fibrin) (Fig. 3).

After two weeks the healing process was completed (Fig. 3).

The lesion, conserved in phormalyne, was sent to the pathologist to make a histological examination: the sample showed limited areas of carbonisation confirming the diagnosis of papilloma.

**Table 2:**
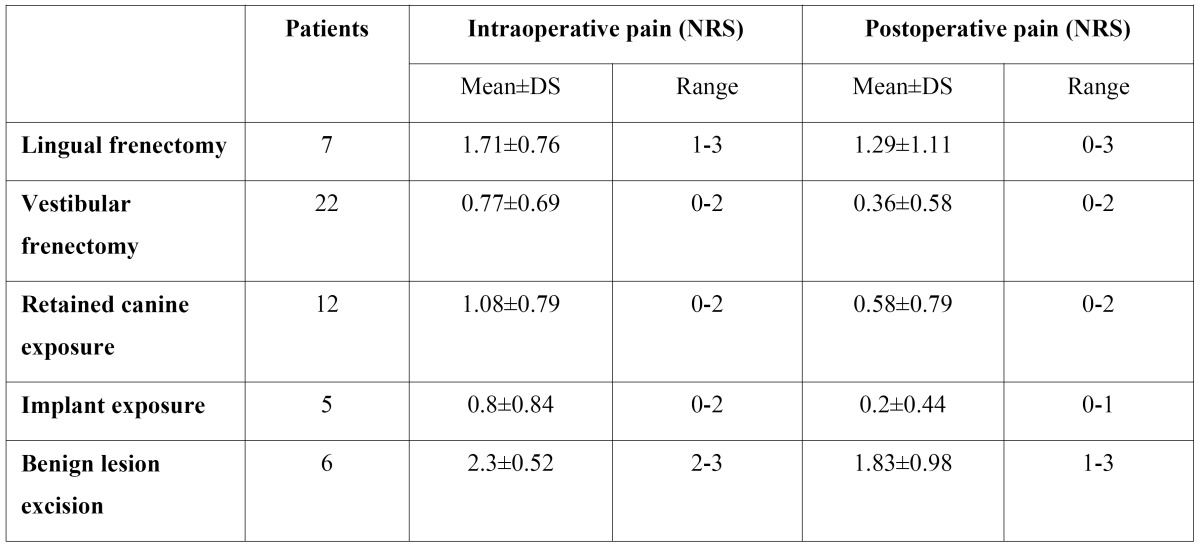
NRS evaluation of intra and postoperative pain for the different types of surgical interventions.

## Discussion

During operations 532 nm wavelength showed to be very efficacious and extremely versatile, although used at low power settings; the day after operation the healing process was good, quick and without complications. The evaluation of intra- and postoperative pain with a NRS allows us to affirm that the patients do not feel a high grade pain, even if the absence of a control group do not permit a comparison with other surgical techniques. 

Patients referred an intraoperative pain between 0 and 3: this is because the anaesthetic procedure was performed only with a topical anaesthetic; the patients referred the feeling of a discomfort but no high grade pain and this may be useful, particularly in children, to avoid the use of local anaesthesia. Postoperative pain, during the 3 days after surgery, had a mean value of 0.8 with a range of 0-3: this allows the patients to follow a normal diet and to have a normal social life avoiding the use of pain killers in the postoperative period. 

The use of wavelength of 532 at low power (1 Watt) for soft tissues surgery of the oral cavity offers a new opportunity to fully exploit the biomodulating effects of laser allowing a good healing process together with an efficacious ablative capability with good pain control and post-operative discomfort.

The use of this wavelength, with the parameters here described, seems to be able to be extended to almost all diseases and tissues of the oral cavity even if, due to the number of patients treated, this study must be integrated with a larger number of cases.
